# The Association of Matrix Metalloproteinase‐1, ‐2, ‐3, ‐7, and ‐13 Gene Polymorphisms With Peri‐Implantitis in an Iranian Population: A Case–Control Study

**DOI:** 10.1002/cre2.70049

**Published:** 2024-11-28

**Authors:** Leila Saremi, Soheil Shahbazi, Mohammad Ebrahim Ghaffari, Saharnaz Esmaeili, Shirin Lotfipanah, Reza Amid, Mahdi Kadkhodazadeh

**Affiliations:** ^1^ Dental Research Center Shahid Beheshti University of Medical Sciences Tehran Iran; ^2^ Department of Biology, Science and Research Branch Islamic Azad University Tehran Iran; ^3^ Dentofacial Deformities Research Center, Research, Institute of Dental Sciences Shahid Beheshti University of Medical Sciences Tehran Iran; ^4^ Department of Epidemiology and Biostatistics, Faculty of Health Qom university of Medical Sciences Qom Iran; ^5^ Department of Biology Education Farhangian University Tehran Iran; ^6^ Iranian Center for Endodontic Research, Research, Institute for Dental Sciences, Dental School Shahid Beheshti University of Medical Sciences Tehran Iran

**Keywords:** alleles, DNA, genotype, matrix metalloproteinases, peri‐implantitis, polymorphism

## Abstract

**Objectives:**

Peri‐implantitis (PI) is the most common biological issue surrounding dental implants. According to current knowledge, the aforementioned complication is not equally distributed across different populations, and gene polymorphisms might be one contributing factor. The current study aimed to examine the association between gene polymorphisms of matrix metalloproteinase‐ (MMP‐) 1, ‐2, ‐3, ‐7, and ‐13 with PI in an Iranian demographic.

**Material and Methods:**

The study's sample included 50 subjects suffering from PI and 89 healthy controls. From each participant, a venous blood sample of 5 cc was obtained, and DNA was extracted. Gene polymorphisms were investigated using restriction fragment length polymorphism polymerase chain reaction (RFLP‐PCR) combined with electrophoresis. Statistical analyses were done using the Pearson chi‐square test, odds ratio, and *t*‐test via SPSS version 28.

**Results:**

The MMP‐3 (‐1171 5A/6A) and MMP‐7 (‐181 A/G) gene polymorphisms were significantly different between the patients with PI and healthy controls (PV < 0.001 and =0.025, respectively). MMP‐1 (‐1607 1G/2G), MMP‐2 (‐1306 C/T), and MMP‐13 (‐77 A/G) gene polymorphisms did not, however, differ in terms of prevalence between the two groups (PV > 0.05). Moreover, the presence of the 6 A allele in the MMP‐3 (‐1171 5A/6A) genotype resulted in a significant decrease in PI risk (PV < 0.001).

**Conclusions:**

Gene polymorphisms in the genotypes of MMP‐3 (‐1171 5A/6A) and MMP‐7 (‐181 A/G) were differential when comparing PI patients and healthy controls of the studied population.

## 1 Introduction

In contemporary dentistry, dental implants are widely used to substitute a single tooth or a group of missing teeth. Despite the 96% survival rate documented for implants, some complications may occur in their surrounding tissues, affecting the treatment's success (Howe, Keys, and Richards 2019). Peri‐implantitis (PI), the most prevalent complication throughout implant dentistry, is a condition in which inflammation has spread beyond the soft tissue boundaries, and ongoing peri‐implant bone loss is evident (Berglundh et al. 2018). According to the literature, approximately one out of every five patients who have received dental implants suffers from PI (Diaz et al. 2022).

PI is a multifactorial disease arising from the interplay between microorganisms' activity and host immunity. The immune response is the factor that gets affected by the genotype of every individual and could explain the varying degrees of disease progression in patients despite having relatively comparable pathological conditions (Dereka et al. 2012). Various cytokines (e.g., TNF‐α and IL‐1β) and enzymes (e.g., matrix metalloproteinases (MMPs)) participate in disease progression during the host's inflammatory response (Saremi et al. 2021; Luchian et al. 2022).

MMPs are enzymes capable of breaking down components of the extracellular matrix (ECM). Microorganisms in the dental plaque stimulate the release of MMPs from host cells, disrupting the balance of these enzymes and their tissue inhibitors (TIMPs). This imbalance potentially causes tissue destruction and periodontal diseases (Checchi et al. 2020). For instance, Zhang et al. have claimed that MMP‐8 fueled the development of periodontitis (Zhang et al. 2018). Likewise, Thierbach et al. (2016) reported higher MMP‐8 levels in the crevicular fluid deriving from PI‐affected implants.

Gene polymorphisms are alterations in the DNA sequence of at least 1% of a specific population, which may affect the gene's expression (Robert and Pelletier 2018). The association of gene polymorphisms and the incidence of periodontal or peri‐implant complications has been investigated for a while. Certain polymorphic genotypes of IL‐10, IL‐1ß, IL‐17, Fc gamma receptors, and tumor necrosis factor‐α may participate in the pathogenesis of PI and chronic periodontitis (Saremi et al. 2021, 2019, 2022; Kadkhodazadeh et al. 2013). Remarkably, significant associations have been found between MMP‐1, 3, and 7 gene polymorphisms and the incidence of chronic periodontitis (Saremi et al. 2023).

Gene polymorphisms affect not only the tissues surrounding teeth but also contribute to the development of peri‐implant disease. Polymorphisms within the promoter region of the MMP‐1, ‐8, and ‐13 genes have been linked to implant loss (Munhoz et al. 2018, 2019). Moreover, MMP‐8 gene polymorphism has been introduced as a possible risk factor for PI (Chmielewski and Pilloni 2023). While considerable evidence supports the association between well‐studied MMP polymorphisms (e.g., MMP‐8) and the incidence of PI (Chmielewski and Pilloni 2023), further investigation into understudied MMPs and their activators is crucial to ascertain their roles in periodontal and peri‐implant diseases definitively. Furnishing clinicians with insights into MMP gene polymorphisms and their association with peri‐implant diseases facilitates cautious patient selection, customization of treatment plans, and prevention of potential complications.

Therefore, this case‐control study was designed to scrutinize the gene polymorphisms of MMP‐1 (‐1607 1 G/2 G), MMP‐2 (‐1306 C/T), MMP‐3 (‐1171 5 A/6 A), MMP‐7 (‐181 A/G), and MMP‐13 (‐77 A/G) among two groups of patients with PI and with healthy peri‐implant conditions in Shahid Beheshti School of Dentistry, Tehran, Iran.

## 2 Materials and Methods

**Figure 1:**
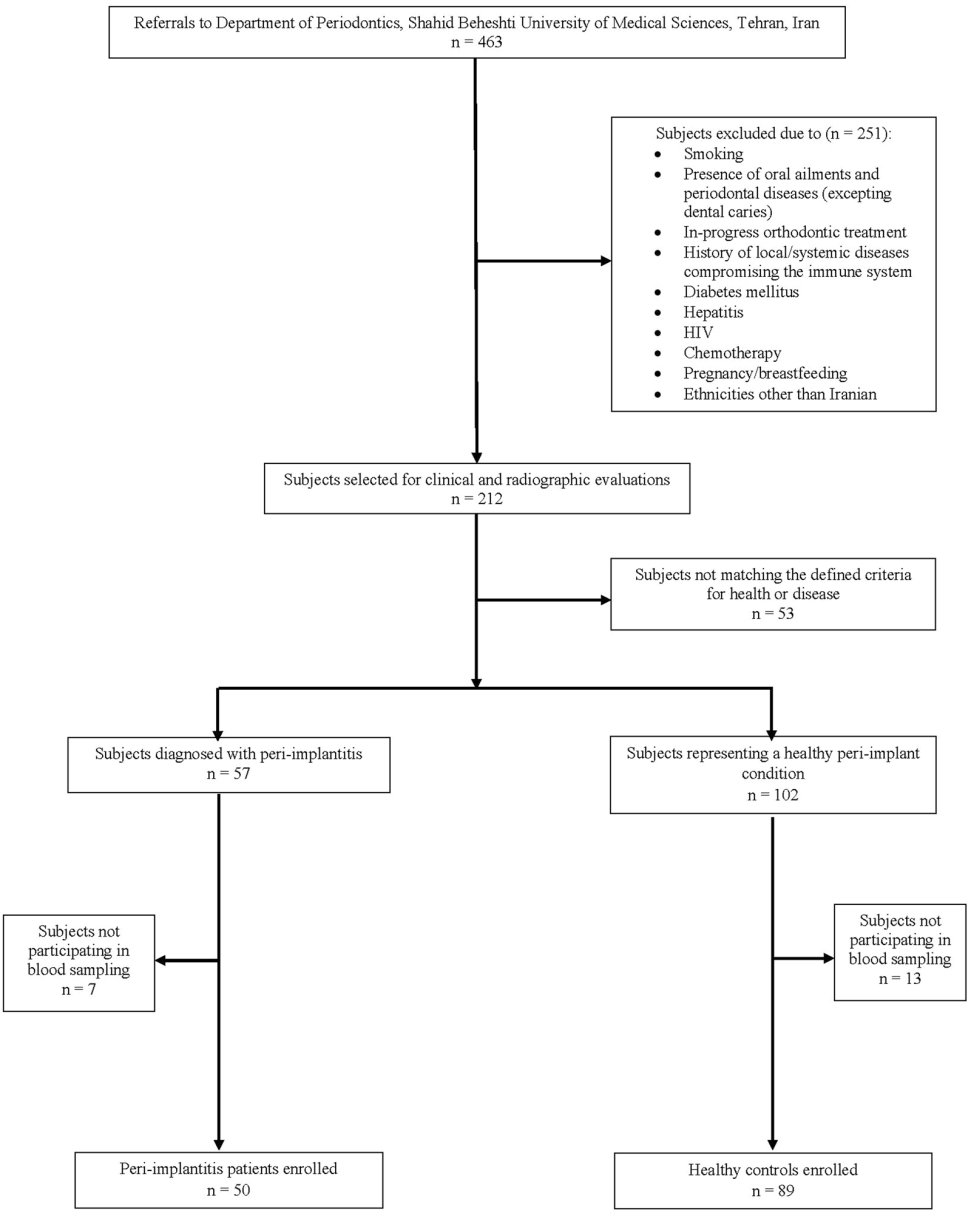
Sample recruitment process.

The current case‐control study received approval from the local ethics committee of Shahid Beheshti University of Medical Sciences (IR.SBMU.DRC.REC.1399.099). Considering the statistical power of 80% and error level of 0.05, as well as an allelic frequency of 0.722 in the control group and 0.37 and 0.352 in the patient group, the minimum sample size for each group was equal to 49, based on a previous study (Saremi et al. 2019). Over a period between May 2020 and April 2022, 50 patients suffering from PI and 89 control patients with healthy peri‐implant conditions were recruited among 463 referrals to the Department of Periodontics, Shahid Beheshti University of Medical Sciences, Tehran, Iran (Figure 1). Their demographic, clinical, and medical information was recorded following the signing of consent forms. The research was conducted in compliance with the Declaration of Helsinki, and its findings were presented following the guidelines outlined in the STROBE checklist.

The following were the criteria for exclusion: smoking, presence of oral ailments and periodontal diseases (excepting dental caries), in‐progress orthodontic treatment, history of local/systemic diseases compromising the immune system, diabetes mellitus, hepatitis, HIV, chemotherapy, pregnancy/breastfeeding, and ethnicities other than Iranian.

PI and peri‐implant health were characterized based on the new classification of periodontal and peri‐implant diseases (Berglundh et al. 2018). Patients of the case group must have been devoid of periodontitis history and had at least one implant loaded for not less than 12 months. PI diagnosis was made according to the following findings: bleeding and/or suppuration on probing, probing depth ≥ 6 mm, radiographic bone loss ≥ 3 mm. Healthy controls with at least one dental implant without signs of inflammation and significant bone loss (< 3 mm) were included in the research if they lacked symptoms or a history of periodontitis. An experienced periodontist (M.K.) did all the mentioned clinical and radiographic evaluations using a long cone parallel peri‐apical technique taken on the screening day.

Patients were chosen based on the previously mentioned criteria, and 5 cc of their venous blood was drawn using venipuncture. Samples were then placed in Falcon tubes lined with EDTA (Merck, Germany). Each patient and their corresponding tube received a unique code. The lab technicians were unaware of the coding system and the group assignments. The collected blood samples were stored at −40°C. Miller's salting‐out method was used to obtain nuclear and mitochondrial DNA, following the protocol provided in the DNA extraction kit (Bioneer Co., Daejeon, South Korea). The DNA concentration was measured with a spectrophotometer, and the samples were then stored at −80°C for future examinations.

Genotyping of the determined MMPs was accomplished using the restriction fragment length polymorphism polymerase chain reaction (RFLP‐PCR) method. The PCR mechanism was employed to amplify the segments containing the desired genes. The gene segments containing the looked‐for polymorphisms were used to make a composition of 1 μL DNA, 0.4 μL deoxyribonucleotide (dNTP) (Fermentas Inc.), 0.5 μL of each primer, 2 μL PCR buffer, 2 μL MgCl_2_, and 0.2 μL Taq polymerase (all constituents were manufactured by Cinnagen Company, Iran). Sterilized distilled water was also added to bring the total volume to 20 μL. The PCR process was done under specific conditions for each gene polymorphism. The yielded DNA fragments were segregated using electrophoresis through 3% agarose gel (Cinnagen Company, Iran). The segments were digested using restriction enzymes at 37°C for a 16‐h period (the process was performed per the manufacturer's instructions). Finally, acrylamide gel electrophoresis (Paya Pajoohesh Pars, Iran) was done at 1000 Volts for 40 min to analyze products. The primers' sequences, PCR settings, restriction enzymes, and DNA fragments are available in Table 1.

**Table 1:** The primers' sequences, polymerase chain reaction (PCR) settings, restriction enzymes, and digestion products.

Gene Polymorphism	Primer Sequence	PCR Settings	Restriction Enzyme	Digestion Products	References
**MMP‐1 (‐1607 1 G/2 G)**	Forward: 5′‐TGACTTTTAAAACATAGTCTATGTTCA‐3ʹ	1 cycle at 95°C for 5 min 30 cycles (95°C for 30 s – 56.5°C for 30 s – 72°C for 30 s) final extension at 72°C for 5 min	Alu I	1 G/1 G: 324 and 121 bp 1 G/2 G: 455, 324, and 121 bp 2 G/2 G: 455 bp	Micheal et al. (2013)
	Reverse: 5′‐TCTTGGATTGATTTGAGATAAGTCATAGA‐3ʹ				
**MMP‐2 (‐1306 C/T)**	Forward: 5′‐CTTCCTAGGCTGGTCCTTACTGA‐3ʹ	1 cycle at 95°C for 5 min 30 cycles (95°C for 30 s – 57.4°C for 30 s – 72°C for 30 s) final extension at 72°C for 5 min	Xspl	CC: 188 bp CT: 188, 162, and 26 bp TT: 162 and 26 bp	Yang et al. (2010)
	Reverse: 5′‐CTGAGACCTGAAGAGCTAAAGAGCT‐3ʹ				
**MMP‐3 (‐1171 5 A/6 A)**	Forward: 5′‐GATTACAGACATGGGTCACA‐3’	1 cycle at 95°C for 5 min 30 cycles (95°C for 30 s – 56.2°C for 30 s – 72°C for 30 s) final extension at 72°C for 5 min	Xmnl	5 A/5 A: 97 bp 5 A/6 A: 120 and 97 bp 6 A/6 A: 120 bp	Lee et al. (2009)
	Reverse: 5′‐TTTCAATCAGGACAAGACGAAGTTT‐3ʹ				
**MMP‐7 (‐181 A/G)**	Forward: 5′‐TGTCCTGAATGATACCTATGAGAG‐3ʹ	1 cycle at 95°C for 5 min 30 cycles (95°C for 30 s – 61.2°C for 30 s – 72°C for 30 s) final extension at 72°C for 5 min	EcoR I	AA: 150 bp AG: 150, and 120 bp GG: 120 bp	Alp et al. (2017)
	Reverse: 5′‐TACTCAGTGGATAAAGGTGTAAGCT‐3ʹ				
**MMP‐13 (‐77 A/G)**	Forward: 5′‐GATACGTTCTTACAGAAGGC‐3ʹ	1 cycle at 95°C for 5 min 30 cycles (95°C for 30 s – 57°C for 30 s – 72°C for 30 s) Final extension at 72°C for 5 min	Bsr I	GG: 436 bp AG: 436, 245, and 188 bp AA: 245 and 188 bp	Ogata et al. (2005)
	Reverse: 5′‐GACAAATCATCTTCATCACC‐3ʹ				

The qualitative data were described using frequency and ratio, while the quantitative data were described by mean and standard deviation. Statistical analyses were done using the Pearson chi‐square test, odds ratio (with a confidence interval of 95%), and *t*‐test via SPSS version 28 (IBM Corp., Armonk, NY) for Windows. *p*‐values smaller than 0.05 were considered statistically significant. Moreover, logistic regression analysis was utilized to assess the significance of various factors that could contribute to the development of PI.

## 3 Results

### 3.1 Demographic Characteristics

The current case–control study included a case group of 50 patients with PI and a control group of 89 healthy patients. The demographic and clinical information of patients is available in Table 2. In terms of age or sex, the two groups did not differ significantly (PV = 0.787 and PV = 0.772, respectively). The plaque index measurements showed a significant difference between the two groups (*p* = 0.032), while the difference in bleeding on probing did not reach statistical significance (*p* = 0.662). The investigated implants were from various systems, including Nobel Biocare (Nobel Biocare, Göteborg, Sweden), SPI (Thommen Medical, Waldenburg, Switzerland), BioHorizons (BioHorizons, Birmingham, MI, USA), and Xive (Densply Friadent, Mannheim, Germany).

**Table 2:** The demographic and clinical information of the subjects.

	Patients (*n* = 50)	Controls (*n* = 89)	*p*‐value
Age (mean ± SD)	41.8 ± 12.2 years	41.2 ± 12.7 years	0.787
Sex			
No. of males (%)	26 (52%)	44 (49.4%)	0.772^a^
No. of females (%)	24 (48%)	45 (50.6%)	
PD (mm) (mean ± SD)	6.81 ± 0.52	1.83 ± 0.68	< 0.001*
RBL (mm) (mean ± SD)	4.44 ± 1.42	0.17 ± 0.11	< 0.001*
BoP (mean ± SD)	0.33 ± 0.12	0.28 ± 0.18	0.662
PI (mean ± SD)	0.62 ± 0.07	0.18 ± 0.06	0.032*

Abbreviations: BoP = bleeding on probing, PD = probing depth, PI = plaque index RBL = radiographic bone loss.

* *p*‐values less than 0.05 are considered statistically significant.

^a^ Analyzed with the Pearson chi‐square test, the remaining values were analyzed with the *t*‐test.

### 3.2 Genotype and Allele Distribution

As demonstrated in Table 3, the disparity in the distribution of genotypes between the case and control groups reached the level of statistical significance in two out of five studied polymorphisms, including MMP‐3 (‐1171 5A/6A) and MMP‐7 (‐181 A/G).

**Table 3:** The distribution of different matrix metalloproteinases (MMPs) genotypes and alleles among patients with peri‐implantitis and healthy controls.

Genotype/Allele		No. patients (%)	No. controls (%)	Pearson chi‐square	*p*‐value	OR	95% CI
**MMP‐1 (‐1607)**							
**Genotype**	1G/1G	17 (34.00%)	38 (42.70%)	1.71	0.425	1	Reference
	1G/2G	24 (48.00%)	41 (46.07%)		0.489	1.31	(0.61, 2.80)
	2G/2G	9 (18.00%)	10 (11.24%)		0.195	2.01	(0.69, 5.85)
**Allele**	1G	58 (58.00%)	117 (65.73%)	1.64	0.200	1	Reference
	2G	42 (42.00%)	61 (34.27%)			1.39	(0.84, 2.30)
	HW‐Chi‐square	0.12	14.67				
**MMP‐2 (‐1306)**							
**Genotype**	CC	8 (16.00%)	7 (7.86%)	2.98	0.225	1	Reference
	CT	22 (44.00%)	36 (40.45%)		0.280	0.53	(0.17, 1.68)
	TT	20 (40.00%)	46 (51.68%)		0.090	0.38	(0.12, 1.19)
**Allele**	C	38 (38.00%)	50 (28.09%)	2.91	0.088	1	Reference
	T	62 (62.00%)	128 (71.91%)			0.64	(0.38, 1.07)
	HW‐Chi‐square	0.49	0.001				
**MMP‐3 (‐1171)**							
**Genotype**	5A/5A	5 (10.00%)	1 (1.12%)	31.43	< 0.001*	1	Reference
	5A/6A	25 (50.00%)	12 (13.48%)		0.649	0.42	(0.04, 3.97)
	6A/6A	20 (40.00%)	76 (85.39%)		0.003*	0.05	(0.006, 0.48)
**Allele**	5A	35 (35.00%)	14 (7.86%)	32.47	< 0.001*	1	Reference
	6A	65 (65.00%)	164 (92.14%)			0.16	(0.08, 0.31)
	HW‐Chi‐square	1.73	32.59				
**MMP‐7 (‐181)**							
**Genotype**	AA	7 (14.00%)	10 (11.24%)	7.35	0.025*	1	Reference
	AG	44 (22.00%)	40 (44.94%)		0.126	0.34	(0.12, 1.27)
	GG	32 (64.00%)	39 (43.82%)		0.772	0.71	(0.40, 3.43)
**Allele**	A	25 (25.00%)	60 (33.71%)	2.29	0.130	1	Reference
	G	75 (75.00%)	118 (66.29%)			1.52	(0.88, 2.64)
	HW^a^ ‐Chi‐square	17.07	43.64				
**MMP‐13 (‐77)**							
**Genotype**	GG	5 (10.00%)	8 (8.99%)	1.16	0.559	1	Reference
	AG	25 (50.00%)	37 (41.57%)		0.901	1.08	(0.32, 3.69)
	AA	20 (40.00%)	44 (49.44%)		0.747	0.73	(0.21, 2.50)
**Allele**	G	35 (35.00%)	53 (29.77%)	0.81	0.369	1	Reference
	A	65 (65.00%)	125 (70.23%)			0.79	(0.47, 1.33)
	HW‐Chi‐square	0.80	35.60				

Abbreviations: CI = confidence interval, MMP = matrix metalloproteinase, OR = odds ratio.

* *p*‐values less than 0.05 are considered statistically significant.

^a^ Hardy–Weinberg equilibrium.

Among patients with PI, the frequency of 5A/6A, 6A/6A, and 5A/5A genotypes of MMP‐3 (‐1171) was 50.00%, 40.00%, and 10.00%, respectively. The corresponding numbers for healthy subjects were 13.48%, 85.39%, and 1.12%, respectively. The frequency of the 6A/6A genotype was significantly greater in the control group (PV = 0.003).

Exploring the MMP‐7 (‐181) genotype distribution among PI patients showed that GG, AG, and AA genotypes were frequent, with rates of 64.00%, 22.00%, and 14.00%, respectively. In the control group, these values were 43.82%, 44.94%, and 11.24%, respectively.

Regarding comparing the alleles of investigated polymorphisms (Table 3), patients with the 6 A allele in their MMP‐3 (‐1171) genotype had a 0.16 times lower risk of PI than homozygous patients with the 1G allele (PV < 0.001). No statistically significant differences were observed in the distribution of other genotypes and alleles.

### 3.3 Logistic Regression Analysis

As shown in Table 4, the logistic regression analysis depicted a significant association between a lower risk of PI and MMP‐3 (‐1171) 6A/6A or 5A/6A genotypes (PV = 0.023).

**Table 4:** Logistic regression analysis of risk factors associated with the incidence of peri‐implantitis.

Predictive factors	Odds ratio (95% CI)	Wald test (*p*‐value*)
Sex	0.87 (0.37–1.85)	0.960
MMP‐1 (‐1607) 2G/2G + 1G/2G	1.45 (0.70–2.97)	0.314
MMP‐2 (‐1306) CT + TT	0.45 (0.15–1.32)	0.138
MMP‐3 (‐1171) 6A/6A + 5A/6A	0.10 (0.01–0.90)	**0.023**
MMP‐7 (‐181) AG + GG	0.78 (0.28–2.19)	0.633
MMP‐13 (‐77) AG + AA	0.89 (0.27–2.88)	0.838

Abbreviation: CI = confidence interval.

* Statistically significant values are presented in bold.

## 4 Discussion

PI is the most frequent complication of dental implants, which may result in implant loss if not managed properly (Rokaya et al. 2020). Therefore, recognizing the factors that may warn the clinician about the risk of disease occurrence in the future would be beneficial. In the current study, the gene polymorphisms of MMP‐1 (‐1607 1G/2G), MMP‐2 (‐1306 C/T), MMP‐3 (‐1171 5A/6A), MMP‐7 (‐181 A/G), and MMP‐13 (‐77 A/G) were compared between Iranian patients with PI and healthy controls. Our findings implied that MMP‐3 (‐1171 5A/6A) and MMP‐7 (‐181 A/G) gene polymorphisms significantly differed between the two groups. MMP‐1 (‐1607 1G/2G), MMP‐2 (‐1306 C/T), and MMP‐13 (‐77 A/G) gene polymorphisms, on the other hand, were not significantly different between the two groups.

The participation of MMPs in the degradation of ECM and, consequently, periodontal destruction has been proved in the current literature (Checchi et al. 2020). As a consequence, marked advancements have been made regarding the potential usage of MMPs for predicting and diagnosing periodontal diseases. For instance, high MMP‐8 levels are blameworthy for periodontal tissue obliteration, making it a powerful diagnostic tool for periodontitis (Hernández et al. 2021). Regarding PI, low levels of MMP‐8 were linked to peri‐implant health, while high levels were related to a higher risk for peri‐implant inflammation (Luchian et al. 2022). In a review by Chmielewski and Pilloni (2023), MMP‐8 is introduced as a diagnostic cytokine representing the highest accuracy and sensitivity.

While the role of MMP‐8 in periodontal and peri‐implant ailments has been extensively studied, there is limited research on the genetic evaluations of MMP‐8 activators, such as MMP‐3 and MMP‐7, in disease progression. MMP‐8 is activated by enzymes such as stromelysin, matrilysin, trypsin‐2, and cathepsin G within the extracellular environment (Johnson, Dyer, and Hupe 1998). MMP‐3, also known as stromelysin‐1, relies on ProMMP‐7 and ProMMP‐8 as substrates, leading to the release of MMP‐7 and MMP‐8 during the activation process (Luchian et al. 2022; Johnson, Dyer, and Hupe 1998). Additionally, MMP‐7 has been identified as an activator of MMP‐8 during the upregulation of MMP‐13 (Dozier, Escobar, and Lindsey 2006). Accordingly, changes in the gene expression of MMP‐8, as well as its activators, can potentially influence a patient's susceptibility to periodontal or peri‐implant diseases.

MMP‐3 levels have been shown to be escalated in advanced periodontitis and introduced as a prognostic factor for periodontitis progression (Soell, Elkaim, and Tenenbaum 2002; Alpagot et al. 2001). Weng et al. (2016) recognized a connection between MMP‐3 (‐1171 5 A/6 A) gene polymorphism and susceptibility to periodontitis. Astolfi et al. (2006) reported that MMP‐3 would be overexpressed in 5 A carriers in case of cell metabolism alteration. This finding may be responsible for lower systemic diseases representing collagenolytic activity (e.g., cancer metastases and rheumatoid arthritis) in chronic periodontitis patients expressing risk alleles. Moreover, genes of MMP‐1 and ‐3 are located in proximity on chromosome 11q22.3, showing to act in collaborations (Li et al. 2012).

According to our results, the MMP‐7 (‐181 A/G) gene polymorphism was another significantly differential factor between the case and control groups. This specific locus is situated within the promoter region of the MMP‐7 gene. Research has indicated that the presence of the G allele in this region is associated with enhanced expression of the MMP‐7 gene (Kesh et al. 2015). Known as matrilysin‐1, MMP‐7 is distinguished as the smallest enzyme in the MMP family due to the absence of the C‐terminal hemopexin domain, which is typical in other MMPs (Chuang et al. 2008). Beyond its role in degrading ECM components, MMP‐7 critically influences various biochemical processes, including the activation, degradation, and shedding of non‐ECM proteins (Ii et al. 2006). MMP‐7 has been asserted to be pivotal in periodontal destruction as well as cancer invasion and metastasis (Chuang et al. 2008; de Oliveira Nóbrega et al. 2016). This enzyme is mainly produced by non‐injured epithelium, in contrast to other MMPs released following injury (Emingil et al. 2006). Kivelä‐Rajamäki et al. (2003) reported a notable elevation in the MMP‐7 level observed in the crevicular fluid of diseased implants compared to healthy ones. A similar elevation occurs in the GCF of patients suffering from periodontitis, according to Checchi et al. (2020). In contrast, the findings of Emingil et al. (2006) show relatively similar levels of MMP‐7 in patients suffering periodontitis, gingivitis, and even healthy subjects. This may indicate that this metalloproteinase comes into play during the primary phases of host defense and does not play a major role in the development of periodontal diseases.

The results of the current research implied that MMP‐1 (‐1607 1G/2G) gene polymorphism did not significantly differ between PI‐affected patients and controls. According to Irshad et al. (2013), the degree of MMP‐1 expression in the fibroblasts of patients with PI is greater than that of healthy subjects, which may contribute to PI pathogenesis. Since TIMPs inhibit the activity of MMPs, diminished levels of MMP‐1/TIMP‐1 complex, which means higher levels of unbound MMP‐1, would indicate disease progression surrounding implants (Ramseier et al. 2016). Opposing our results, Rutter et al. (1998) concluded that an SNP at −1607 bp in the MMP‐1 promoter region induces more aggressive ECM degradation. This higher degradation may explain the higher rate of periodontal destruction. Moreover, Leite et al. (2008) showed that the presence of guanine at the −1607 location within the MMP‐1 gene might be linked to implant failure.

In terms of allele frequency, we found that the presence of a polymorphic allele in the DNA sequence of MMP‐1 (−1607) would not affect the risk of PI. In opposition to this finding, Cao et al. (2006) reported that subjects with the 2G allele in their MMP‐1 gene promoter region of −1607 bp appeared to be about three times more likely to manifest severe chronic periodontitis. De Souza et al. (2003) also concluded that patients carrying the 2G allele in their MMP‐1 gene seem roughly twice as likely to suffer from severe periodontitis. According to Cao et al. (2010), the presence of the 2G allele in the MMP‐1 (−1607) genotype causes enhanced expression of this enzyme in response to IL‐1β stimulation. In addition, Li et al. (2012) identified the MMP‐1 (−1067) 2G allele as a protective element against chronic periodontitis.

The influence of MMP‐2 (−1306 C/T) gene polymorphism on the incidence of PI was found to be insignificant in the current study. MMP‐2 is capable of degrading Type I, II, III, and IV collagen in conjunction with stimulating cell proliferation and angiogenesis. In conclusion, MMP‐2 takes part in tissue destruction and tissue repair simultaneously (Luchian et al. 2022; Hsiao et al. 2016). Figueiredo et al. (2020) denied any significant alteration in the level of MMP‐2 in PI patients. On the other hand, Kensara et al. (2021) and Che et al. (2017) found a significantly higher level of this enzyme in PI sites. Replacement of the C allele with T in the −1306 location has been shown to decrease the promoter activity of the MMP‐2 gene alongside reducing cancer susceptibility (Hsiao et al. 2016).

Our findings denied any significant difference between patients with PI and healthy subjects regarding MMP‐13 (‐77 A/G) gene polymorphism. Gursoy et al. (2013) reported a greater salivary expression of MMP‐13 in cases suffering from generalized periodontitis. Furthermore, Sorsa et al. (2006) claim that MMP‐13 contributes to the deterioration of attachment loss and the extension of periodontal pockets. Regarding PI, Borsani et al. (2005) observed strong MMP‐13 immunolabelling in the epithelial layer but only a weak presence in the connective tissue. In a healthy peri‐implant site, however, this enzyme was weakly labeled only in the connective tissue.

During the sample recruitment phase, a specific set of inclusion criteria was applied, centered on parameters such as probing depth, bleeding on probing, and bone loss. However, the employment of diverse diagnostic criteria across various studies has led to a diminished generalizability of the results, thereby hindering the achievement of a unified outcome. Among the diagnostic criteria utilized is the implant success index (ISI), which is a simple scoring method emphasizing quantitative over qualitative assessments. It can be employed at baseline and follow‐up visits to compare the outcomes of a specific PI treatment. Its adaptability extends to compatibility with various implant types, whether bone level or tissue level (Kadkhodazadeh and Amid 2012). The ISI scoring system has exhibited a high level of acceptability among implant experts, attributed to its ability for early lesion detection, minimal overestimation, quantitative manner, and comprehensiveness (Kadkhodazadeh et al. 2012). It has manifested superior reproducibility compared to simpler classifications due to its more detailed evaluation approach (Abrishami et al. 2014). Nevertheless, further research involving larger sample sizes is required to reach more conclusive results about the applicability of the ISI scoring system in daily practice. For the enhancement of comparability and applicability of research outcomes pertinent to peri‐implant diseases, adherence to the most recent consensus report of world workshop in subsequent studies is recommended (Berglundh et al. 2018).

These findings could enhance clinicians' ability to assess the risk of patients needing implant placement. Implant treatment planning traditionally considers factors such as age, gender, systemic health, and smoking status; however, a deeper understanding of gene polymorphisms could enable personalized treatment strategies based on a patient's genetic susceptibility to peri‐implant diseases. While routine genetic testing for all patients may not be feasible or cost‐effective, analyzing gene expression products, such as proteins in saliva or GCF, offers an alternative screening method (Katsiki et al. 2021). Identification of genetic risk factors may prompt clinicians to adjust treatment protocols, including modifying plaque control techniques, reducing maintenance intervals, employing antimicrobials, or selecting specific implant designs to prevent complications. For example, MMP gene polymorphisms might make a clinician use MMP inhibitors to treat or prevent PI (Honibald et al. 2012). The pathogenesis of PI is not limited to plaque accumulation and local host responses; genetic factors can significantly influence the outcome, potentially leading to the failure of even the most ideally placed standard dental implants.

In terms of limitations, genetic studies would result in a more accurate outcome if performed prospectively with a large sample size. Unfortunately, this was not feasible in the current clinic‐based study. It should be borne in mind that multiple confounding factors can influence implant success rate, such as plaque control skills, maintenance interval, socioeconomic status, and implant function time (Chatzopoulos and Wolff 2018; Derks and Tomasi 2015). Hence, further studies are required to gather more information in this regard and assess the effect of various risk factors/indicators on PI incidence. Another limitation that may be common among PI studies is the inconsistent characteristics for diagnosing the disease. Therefore, presenting a unit and comprehensive conclusion through reviewing conducted studies may be challenging. It is noteworthy that reputable university clinics, such as Shahid Beheshti Dental School, attract referred patients from diverse geographic regions. As a consequence, the source of sample population may not be comparable in all aspects (e.g., neighborhood), leading to different admission rates for cases and controls. Thus, recruiting samples exclusively from the same institution does not entirely eliminate the potential for selection bias (Sutton‐Tyrrell 1991). Since the majority of genetic research around the role of MMPs in PI is focused on MMP‐8 and MMP‐9, exploring other MMPs seems crucial for future studies. Moreover, exploring the association of MMPs' genetic variations with the peri‐implant response to treatment would be an interesting topic for future research.

## 5 Conclusions

Regarding the studied population, significant differences between PI patients and periodontally healthy subjects regarding MMP‐3 (‐1171 5A/6A) and MMP‐7 (‐181 A/G) gene polymorphisms were observed. However, the relationship between MMP‐1 (‐1607 1G/2G), MMP‐2 (‐1306 C/T), and MMP‐13 (‐77 A/G) gene polymorphisms and PI risk did not reach statistical significance. In addition, the 6 A containing genotypes of MMP‐3 (‐1171) were associated with a lower risk of PI. Clinicians are suggested to consider genetic variations within each population before periodontal treatment planning.

## Author Contributions

Leila Saremi, Reza Amid, and Mahdi Kadkhodazadeh have been involved in the conception and design of the study. Leila Saremi, Soheil Shahbazi, and Shirin Lotfipanah have been involved in data acquisition. Leila Saremi and Mohammad Ebrahim Ghaffari have been involved in data analysis and interpretation. Saharnaz Esmaeili, Soheil Shahbazi, and Shirin Lotfipanah have been involved in drafting the article. Leila Saremi, Reza Amid, and Mahdi Kadkhodazadeh have been involved in revising the article and final approval of the version to be published.

## Ethics Statement

The current case–control study received approval from the local ethics committee of Shahid Beheshti University of Medical Sciences (IR. SBMU. DRC. REC.1399.099).

## Consent

Written consent forms were signed by all patients.

## Conflicts of Interest

The authors declare no conflicts of interest.

## Data Availability

The data that support the findings of this study are available from the corresponding author upon reasonable request.
